# Fusion of Visible and Thermal Descriptors Using Genetic Algorithms for Face Recognition Systems

**DOI:** 10.3390/s150817944

**Published:** 2015-07-23

**Authors:** Gabriel Hermosilla, Francisco Gallardo, Gonzalo Farias, Cesar San Martin

**Affiliations:** 1 Escuela de Ingeniería Eléctrica, Pontificia Universidad Católica de Valparaíso, Valparaíso 2362804, Chile; E-Mails: francisco.gallardo.s@mail.pucv.cl (F.G.); gonzalo.farias@ucv.cl (G.F.); 2 Department of Electrical Engineering, University of La Frontera, Temuco 4811230, Chile; E-Mail: cesar.sanmartin@ufrontera.cl

**Keywords:** face recognition, fusion descriptors, genetic algorithms, visible and infrared spectrum

## Abstract

The aim of this article is to present a new face recognition system based on the fusion of visible and thermal features obtained from the most current local matching descriptors by maximizing face recognition rates through the use of genetic algorithms. The article considers a comparison of the performance of the proposed fusion methodology against five current face recognition methods and classic fusion techniques used commonly in the literature. These were selected by considering their performance in face recognition. The five local matching methods and the proposed fusion methodology are evaluated using the standard visible/thermal database, the Equinox database, along with a new database, the PUCV-VTF, designed for visible-thermal studies in face recognition and described for the first time in this work. The latter is created considering visible and thermal image sensors with different real-world conditions, such as variations in illumination, facial expression, pose, occlusion, *etc.* The main conclusions of this article are that two variants of the proposed fusion methodology surpass current face recognition methods and the classic fusion techniques reported in the literature, attaining recognition rates of over 97% and 99% for the Equinox and PUCV-VTF databases, respectively. The fusion methodology is very robust to illumination and expression changes, as it combines thermal and visible information efficiently by using genetic algorithms, thus allowing it to choose optimal face areas where one spectrum is more representative than the other.

## 1. Introduction

Face recognition in the visible spectrum is a well-known field of research with many years of study using low-cost cameras. Many publications are available, containing smart and sophisticated algorithms. This field has seen steady growth due to its security applications, as well as other types of applications, such as access permission and even identity control. However, one of the main problems that researchers must overcome is the diminished recognition ability of algorithms as a result of variations in the intensity of illumination in the image. In a real application, where intensity of illumination can vary due to factors, such as the time or weather, face recognition with visible imagery may no longer be robust (*i.e.*, low recognition rates).

A solution to the problem caused by lighting in visible imagery is to use images acquired in the infrared (IR) spectrum (specifically, the thermal spectrum, 8–12 µm), because the images obtained in this spectral range remain invariant to lighting changes that may occur in the visible spectrum [1]. This is possible because thermal cameras capture the infrared radiation emitted by the face instead of the reflected light, following Stefan Boltzmann’s law. The estimation of the temperature of a body is a process called thermography [2], in which it is not necessary to make contact with the surface of the body. 

A disadvantage of the IR spectrum is that the energy received by the IR camera not only depends of the measured body, but also on the sum of the energy components of the different elements of the scene captured by the camera. The elements of the scene that are captured are: the atmosphere, the background and the object to be measured. The variation of these components could affect the temperature estimation of the measured body, faces in this case, which is a problem in face recognition. The thermal face images also have undesirable variations due to changes in ambient temperature, alterations of the metabolic processes of the subjects, camera susceptibility to extrinsic factors and variable sensor responses over time when the camera is working for long periods of time [3,4].

Based on the advantages and disadvantages of both spectra, it is possible to create a face recognition system based on the fusion of the visible and thermal domain, achieving a high recognition rate. The aim of this article is described in the following three points: (1) the article proposes a new fusion system that combines visible and thermal features obtained from the most current local matching descriptors by maximizing face recognition rates through the use of genetic algorithms (GA, [5,6]); (2) the article compares and studies current face recognition methods and classic fusion approaches against the proposed fusion technique; and (3) the PUCV-VisibleThermal-Face (PUCV-VTF) database is described for the first time for face recognition, as it was designed for visible-thermal studies in face recognition. The main idea of the fusion method is to maximize face recognition rates using genetic algorithms, which improves recognition rates using visible and thermal descriptors obtained from the newest and best methods currently used in the literature.

The PUCV-VTF database contains thermal and visible images acquired with two different sensors and allows us to study the fusion of these images. The visible images were acquired with a PS3Eye camera, 640 × 480 pixels, while the thermal images were acquired with an FLIR Tau 2 640 camera, with a resolution of 640 × 512 and 14 bits. 

This study includes five local matching methods obtained from different comparative studies of visible and thermal methods [7,8,9,10,11,12], such as local binary pattern (LBP) [13], the Weber law descriptor (WLD) [14] and the Gabor jet descriptor (GJD) [11], due to their high performance. In addition, new methods that work in the visible spectrum, such as local derivative patterns (LDP) [15] and HOG descriptors [16], are also included due to the high performance seen in recent studies [15,16,17]. To perform the fusion method and based on results obtained previously [7,8,12], we choose two local descriptor methods, the LDP and LBP descriptors, to perform the combination. Thus, the genetic algorithm combines both descriptors in order to maximize the recognition rate of the entire face recognition system.

The main differences between our proposed fusion method and other fusion studies [18,19,20] are presented in the following paragraph. In [18,19], standard appearance-based methods are used together with genetic algorithms for the analysis and fusion of visible and thermal data. The studies use the classic eigenfaces approach for fitness evaluation, in which it is difficult to describe the face efficiently, unlike with our method, which uses new approaches for face description. In addition, our way of performing the fusion is different, because we use only 64 weights for the fused descriptor against the 100 principal components in [18], for example. In [20], the fusion of visual and thermal images using the discrete wavelet transform (DWT) domain is described. The results of the experiments demonstrate that the fusion method is effective in terms of visual quality compared to conventional visible and thermal approaches, but it depends on glasses being present in the image. In our work, the selected face recognition descriptors are robust to the occlusion produced by glasses [7]. In [21,22], genetic algorithms are not used to perform the fusion; however, those articles use methods based on data fusion and decision fusion. In [21], the fusion algorithm is designed to detect and replace glasses with an eye template in the case of thermal images, and FaceIt, a commercial face recognition software, is used to evaluate the fusion algorithm. In [22], the advantages of combining thermal and visible face recognition are analyzed, and recognition is achieved using a k-nearest neighbor classifier by principal component analysis (PCA). Finally, [23] uses a fusion method based on image fusion using the entropy information with a genetic algorithm. The study does not perform face recognition; however, it shows a way to efficiently combine visible and thermal features to be used in a face recognition system.

The proposed fusion methodology and the current visible-thermal methods will be evaluated using the Equinox database [24] and a new database named PUCV-VTF, which has been created to compare recognition rate results and to validate the fusion method. The Equinox database was used in [7,9,10], and it consists of face images in the visible, long-wave infrared (LWIR), mid-wave infrared (MWIR) and near-wave infrared (NWIR) spectra. For this study, only the visible and LWIR are used with six gallery sets and nine test sets for both spectra. In addition, we will compare the face recognition results obtained using the fusion approaches from [18,19,20] against the proposed methodology using the Equinox database. The PUCV-VTF database consists of face images in the visible and long-wave infrared spectra (thermal images), taken at the same time. We will create five sets, using a frontal set as the gallery and the others as test sets. Specifically, in our experiments, we use the Equinox database to generate the weights with the genetic algorithm for the fusion method, and we then evaluate the fusion method using the PUCV-VTF database.

This paper is structured as follows: Section 2 describes the current visible and thermal methods used in face recognition systems. The proposed methodology of visible-thermal descriptor fusion using a genetic algorithm is explained in Section 3. The visible and thermal databases and the image sensors used in this study are presented in detail in Section 4. Section 5 shows the experiments to obtain the optimal population of the fusion scheme and the validation of the fusion methodology. Finally, the main conclusions are presented in Section 6.

## 2. Visible-Thermal Methods

As was mentioned in the previous section, we selected five current methods used in the literature to generate face recognition systems in the visible and thermal domains. The methods were chosen considering high performance and requirements, such as working in real time, using only one image per person in a gallery for face recognition matching (no training stage) and their high rates in comparative studies [7,8,9,10,11,12].

### 2.1. LDP Histograms

The local derivative pattern of n-th order was first proposed in [15]. The method obtains micropatterns that use the information contained in directional derivatives of (n − 1) order of the original image. The method uses different ***α*** directions to obtain a descriptor: 0°, 45°, 90° and 135° only, since 180°, 225°, 270° and 315° are implicitly contained in the directions used. The micropatterns are obtained using a neighborhood of 8 pixels around a central pixel, taken from the derivative image in the ***α*** direction to extract the information using a comparative function. To give a holistic feature to LDP, the image is divided into square regions where histograms with 256 bins are computed. The information taken from all of the directions for every region is concatenated for the final LDP n-th order descriptor of the original image.

### 2.2. LBP Histograms

The local binary pattern was originally proposed in [13]. It was first designed for the description of textures and was then adapted to face recognition. The method compares the intensity differences between the central pixel and its neighborhood in a 3 × 3 region to generate a binary code. One advantage of this method is its computational efficiency; this is important in image processing in real time. With this method, three levels of locality are obtained: pixel level (information about patterns obtained pixel to pixel), regional level (where groups of pixels give information about small regions of the face) and the holistic level of locality. A global description of the face is obtained concatenating the regional LBP extracted features using histograms by region. 

### 2.3. Histograms of Oriented Gradients

Histograms of oriented gradients, proposed in [16], were used to obtain a human descriptor for pedestrian applications. The HOG algorithm uses the information contained in both the magnitude and orientation of the gradients presented in the original image. The face image is divided into *mxm* regions, where the histograms are calculated with 9 bins using the information on magnitude and orientation. These histograms are concatenated to obtain the final HOG descriptor of the original image, taking half of the precedent region in both directions to overlap the face information. 

### 2.4. Weber Local Descriptor

The Weber local descriptor was first proposed in [14] and is based on the fact that the human perception of a pattern does not depend solely on a stimulus change, but also on its intensity. Specifically, WLD consists of two-dimensional histograms: differential excitations and orientations. The differential excitation component is a function of the radius between two terms: the first term is the relative difference in intensity of the current pixel against its neighbors, and the second term is the intensity of the current pixel itself. The orientation component is the orientation of the gradient of the current pixel. Thus, given an image, both components are used (differential excitation and orientation) to build and concatenate the WLD histogram.

### 2.5. Gabor Jet Descriptor

Gabor filters are used in face recognition because of their similarity to cell behavior in visual perception and of their robustness to change in illumination. The filters extract characteristics by means of the selection of frequency, orientation and scale. The jets are calculated at different points on a grid, and then, the Borda count method is applied to obtain a ranking and, thus, to generate the recognition. The implementation for this study was based on [11], in which several local feature representations, classification methods and combinations of classifier alternatives were compared. That system uses Gabor jet descriptors as local features, which are uniformly distributed over the images, one wavelength apart. In each grid position of the test and gallery images and at each scale (multiscale analysis), the Gabor jets are compared using normalized inner products. These results are combined using the Borda count method. 

To summarize, we used five local descriptor-based methods, LDP, LBP, HOG, WLD and GJD, to perform the face recognition. For LDP, LBP and HOG, we used different divisions of the image to give them a holistic feature in partitions of 20 × 4, 8 × 4 and 16 × 16 regions, in order to find the best partition regions in our analysis. For WLD, we consider the results obtained from [7], which uses only 20 × 4 partitions. The recognition for the local descriptor method is performed using a nearest neighborhood with one of the three following similarity measures: Euclidean distance (EU), histogram intersection (HI) and chi squared (X2). For the case of the Gabor jet descriptor, the image is not partitioned, and the Borda count is used to compute the matching for the face recognition. In addition, the following notation is used to name each method and variation in this study: A_B_C, A describes the name of the method (LBP, HOG, GJD, LDP n-th and WLD), B shows the similarity measure (EU, HI or X2); C denotes the number of regions used (80 for 20 × 4, 32 for 8 × 4 and 256 for 16 × 16). In the case of GJD, only the Borda count is used, and the image is used as a whole, so its notation is simply “GJD”.

## 3. Proposed Method: Visible-Thermal Face Descriptor Fusion by Genetic Algorithms

Genetic algorithms have been used since 1975 by applying Darwin’s theory [5,6], and their use is inherently associated with maximizing or minimizing the result of a fit function by means of natural selection and mutation genetics, thus comprising a robust method of finding solutions that is not mathematically guided. 

**Figure 1:**
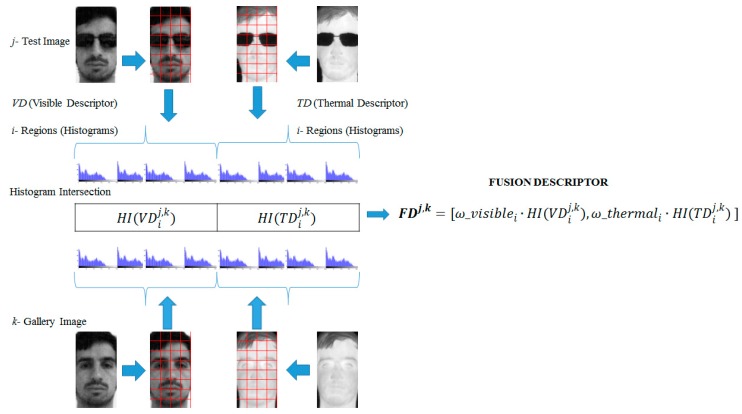
Fusion scheme process.

We propose the use of a fusion technique based on genetic algorithms, which combines the most relevant information from visible and thermal images with image descriptors. Our genetic algorithm finds a genetic code, which consists of weights for the visible and thermal descriptors to maximize the recognition rate as an objective function. The fusion method is performed by taking an input pair of test images (visible and thermal) to be compared against a pair in the gallery set, and following this, two face recognition descriptors are applied: the visible descriptor (VD) and the thermal descriptor (TD) (any local descriptor can be chosen). To combine the descriptors, histogram intersection is used as the similarity measure to perform the fusion. Figure 1 shows the fusion method. 

Combinations of image descriptors were selected based on the results of [7,12,15]; for the visible spectrum, two descriptors were chosen: the third order LDP descriptor (LDP3), with 256 divisions in the image, and the commonly-used LBP; for the thermal case, only the LBP descriptor with 32 regions was used, as it is simple and obtains high results. The fusion method combinations are therefore based on: LDP-LBP and LBP-LBP for the visible and thermal descriptors. In addition, note that the LDP descriptor for visible image uses 256 regions, and the thermal image uses 32 regions; thus, to perform the fusion, we take groups of 8 sectors for the visible LDP descriptor. Therefore, the length of the genetic code given by the GA is of 64 weights, where the first 32 correspond to visible weights and the following 32 to thermal weights. Once the recognition rate was obtained, our fusion scheme maximized this recognition rate using the weights obtained from the GA process.

### 3.1. Proposed Genetic Algorithm for Fused Descriptors

Basically, a genetic algorithm (GA) searches for the optimum result of an objective function. First, an initial population of possible solutions has to be created and then evaluated using the objective function (also known as a fitness function) obtaining the fitness value of each individual in the population. After the initial population is established, several steps put the GA into a loop algorithm, which can be stopped by different criteria, such as an expected fitness value or number of iterations. This basic method also involves the selection of individuals as parents, crossover and mutation and finally reinsertion into the population. 

The starting point of our GA was the choice of a function to maximize fitness, in our case the recognition rate. The GA assigned weights *ω* = [*ω_visible ω_thermal*] to the partitions of each image (visible and thermal), which represent the genetic code to be optimized. The process considers the face recognition results obtained from each test image *j*, gallery image *k* and the image partitions *i* of the regions of the histogram of the visible descriptor and the thermal descriptor (see Figure 1). Mathematically, the vector fusion descriptor (*FD*) for the test image *j* and the gallery image *k* can be described by the following Equation (1):

(1) $${\text{FD}}^{j,k}=\left[\omega \_{\text{visible}}_{i}\cdot \text{HI}({\text{VD}}_{i}^{j,k}\right),\;\omega \_{\text{thermal}}_{i}\cdot \text{HI}({\text{TD}}_{i}^{j,k})\;]$$

where *i* is the number of regions of the image, *VD* is the visible image descriptor, *TD* is the thermal image descriptor and *ω_visible* and *ω_thermal* are the weights of the population to be optimized. The histogram intersection function *HI(·)* combines the descriptor regions from the test image *j* and gallery image *k* and is given by $HI(S^{j,k})=\sum_{w}\text{min}(S_{w}^{j},S_{w}^{k})$. 

Once the objective function was chosen and the nature of the genetic code was established, the GA was run with given gallery and test sets, aiming to find the best recognition performance between them using the GA with the following steps. 

Initial population:

1. An initial population of 100 genetic codes was randomly made with 64 weights in the interval [0, 1]. The initial population had complementary weights for visible and thermal regions.
2. The genetic code was applied to the descriptor database of gallery and test images using Equation 1. The similarity value was then calculated using $SV^{j,k}=\sum_{i=1}^{64}FD_{i}^{j,k}$.
3. The face recognition was performed, giving the maximum value of the similarity value (SV) to each test image j, and the recognition rate was then calculated using the correctly-recognized faces. Note that the fitness value of each genetic code is the recognition rate, which was then optimized.

New offspring generation:

4. Two genetic codes were selected as parents, using the roulette method [5,6].
5. The parents were crossed with a probability of 25% at each point, obtaining two offspring (the weights of visible and thermal were no longer complementary).
6. The offspring was mutated at three random points with a probability of 25%.
7. The fitness values of both offspring were calculated as for the initial population (Steps 2–3).
8. If the fitness value of a given offspring was greater than the lowest fitness value in the population, then a random genetic code was replaced, otherwise it was discarded.
9. If the iteration number was under 100,000, Step 4 was repeated.
10. End.

The genetic algorithm process may take a considerable amount of time to obtain the genetic code. However, the GA process is performed only to find the best optimal weights to obtain the best genetic code, similar to an offline stage. After this process, the face recognition is performed with the fusion method using only the best code obtained. 

## 4. Visible and Thermal Databases

To carry out the fusion analysis, the Equinox database and the PUCV-VTF database were used. A brief description of the databases is given in the following sections. It is important to mention that the PUCV-VTF was created for this research and considers images in both the visible and thermal spectra of 76 individuals and different face conditions, such as emotions, gestures and artefacts (glasses).

### 4.1. Equinox Database Description

**Figure 2:**
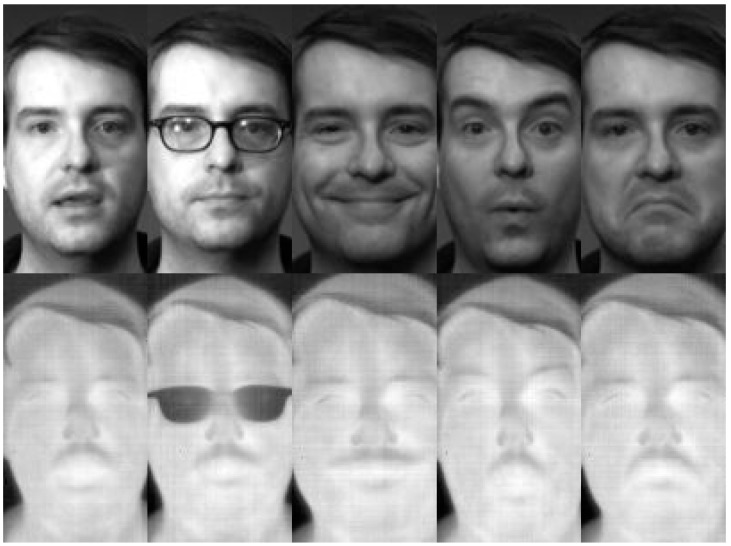
Equinox database examples.

The Equinox database [24] has been used in several studies to compare and evaluate face recognition methods. Thus, it allows direct comparison between methods implemented previously, such as PCA, Independent Components Analysis (ICA), LBP, WLD, GJD, kernel PCA (KPCA) and kernel Fisher linear discriminant (KFLD) [9,10,19,25]. The Equinox database is often used to study the performance of different face recognition algorithms using thermal imaging (8–12 microns). The database consists of several sets of frontal images taken in both visible and infrared spectra at the same time, totaling 18,629 pictures for each spectrum with grey scale with a size of 240 × 320 pixels. The gallery set contains images from 81 different subjects, which were obtained under different lighting (frontal, left lateral and right lateral), three different expressions (smile, frown, surprise), while the test sets have more images per subject (see Table 1). Subjects who use glasses are captured twice, with and without their glasses. The images were processed to be 150 pixels in height and 81 pixels in width, with the eyes horizontally centered, aligned and with a distance between them of 42 pixels, showing only the face of the subjects (see the examples in Figure 2).

For the evaluation of the methods used in this study, 15 sets of images were established with the following criteria (taken from [12]). All subjects have images in each set (visible and thermal); for gallery sets, there is only one image per subject (81 images per set); the test sets have different sizes, as detailed in Table 1. Thus, six gallery sets were established (VA, EA, VF, EF, VL, EL) and nine test sets (VA, EA, VF, EF, VL, EL, VG, EG, RR), see details in Table 1.

**Table 1:** Equinox Database Description. The quantity of images the test sets is detailed in the table.

Sets	Description	Subjects	Illuminations	Image Number
VA EA	Vowel Frames Expressions frames	All Subjects All Subjects	All illuminations All illuminations	729 images 729 images
VF	Vowel frames	All Subjects	Frontal illumination	243 images
EF	Expressions frames	All Subjects	Frontal illumination	243 images
VL	Vowel frames	All Subjects	Lateral illumination	486 images
EL	Expressions frames	All Subjects	Lateral illumination	486 images
VG	Vowel frames	Subjects Using Glasses	All illuminations	324 images
EG	Expressions frames	Subjects Using Glasses	All illuminations	324 images
RR	Random 500 frames	Chosen at random	All illuminations	500 images

### 4.2. PUCV-Visible Thermal-Face Database Description

The PUCV-VTF database [26] was developed to contribute to the research community interested in face recognition. The visible images were acquired with a PS3Eye camera [27], with the following specifications: image resolution of 640 × 480 pixels, field of view between 56° and 75° and a frame rate of 120/60 frames per second.

Thermal images were acquired with a FLIR Tau 2 camera [28], with the following specifications: image resolution of 640 × 512 pixels and 14 bits, field of view between 56° and 69°, frame rate of 30 frames per second, thermal sensibility of 50 mK and a spectrum range between 7.5 and 13.5 µm.

**Figure 3:**
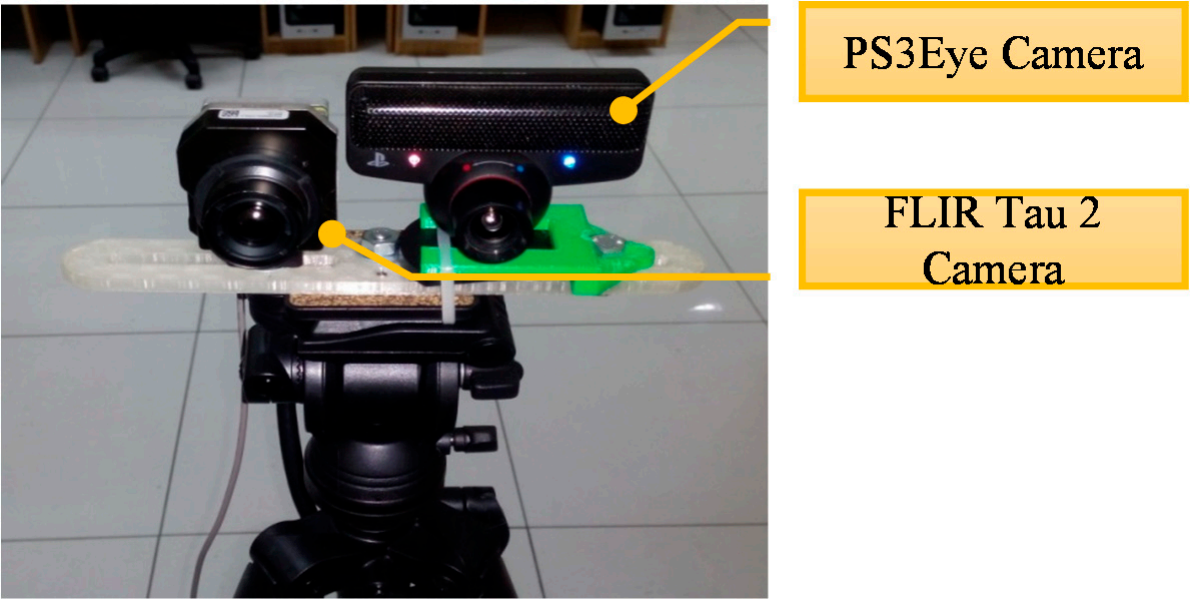
Physical setup of the PUCV- VisibleThermal-Face (VTF) database acquisition system.

The physical setup is shown in Figure 3. This consists of a tripod where the two sensors (FLIR Tau 2 and PS3 Eye) are positioned with a separation of 6.5 cm. These cameras are at a height of 115 cm from the floor and at a distance of 91.5 cm from the background. The background is a white wall, which was chosen to avoid thermal and visible changes that might affect the acquisition of the images. 

**Figure 4:**
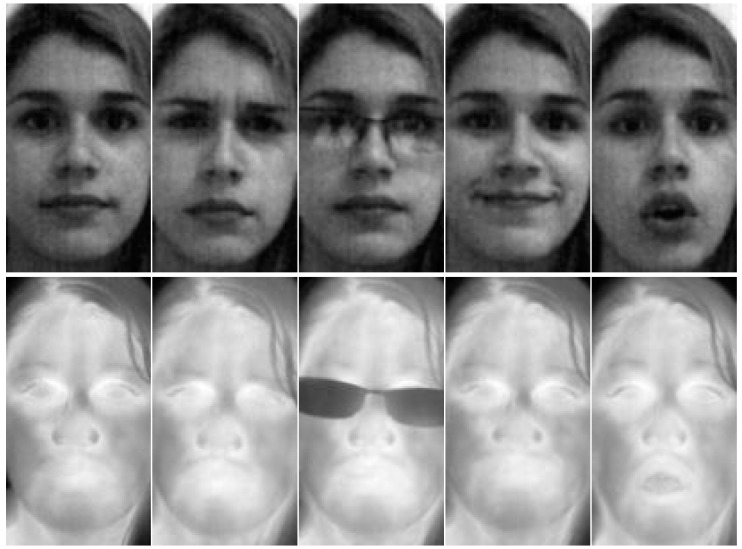
PUCV-VTF database examples.

The database includes 76 individuals with five subsets, in a total of 12,160 images in both visible and thermal spectra. The sets comprise normal faces (frontal condition), frown, glasses, smile and vowel. Only one image per subject is used to create the sets. The normal set was considered as the gallery, as it is neutral, while the rest were used as test sets. The characteristics of the sets are the following: normal faces indicate a serious pose of the individual; frown indicates anger; glasses are images of the individual using transparent or dark glasses at random; smile shows some grinning; and vowel faces are images of the individuals articulating a vowel of their choice. We use the following notations for each set: Nml for normal, Frn for frown, Gls for glasses, Slm for smile and Vwl for vowel (see the examples in Figure 4).

All of the images were cropped and aligned according to the coordinates of the eyes, which were marked manually. The length and proportion of the distance between the eyes and the borders were made in line with the Equinox database (81 × 150 pixels with 42 pixels between the eyes). The visible and thermal images were normalized using the following Equation (2), which consists of applying a linear mapping to the pixels intensity values in the range [N_min_, N_max_]:

(2) $$I_{norm}\left(i,j\right)=\frac{I\left(i,j\right)-I_{\text{min}}}{I_{\max}-I_{\text{min}}}\left(N_{max}-N_{min}\right)+N_{min}\;\forall \left(i,j\right)\in \Omega$$

where *I_min_* and *I_max_* are the minimum and maximum values in the image: $I_{min}=min_{\left(i,j\right)\in Ω}I\left(i,j\right)$ and $I_{max}=max_{\left(i,j\right)\in Ω}I\left(i,j\right)$. In the experiments, the value range of [0,255] is used.

## 5. Experiments

In order to evaluate the proposed fusion methodology using GA, the face recognition experiments were performed using two databases: Equinox and PUCV-VTF. The first experiment consists of comparing face recognition results from the fusion method against other face recognition methods and the fusion results obtained from [18,19,20], thus obtaining the best configuration of our methodology and the optimal population to achieve high recognition rates. The GA was run in order to obtain the best genetic codes for the fusion method. After the experiment, the optimal population was selected. This is then validated with the PUCV-VTF database, comparing the results with the visible and thermal algorithms used in the literature.

### 5.1. Experiment 1: Optimal Population in the Equinox Database

The main idea of this experiment is to obtain the best genetic codes with the GA process for the proposed fusion method described in Section 3. The fusion methods used 2 variants composed of the combination of visible and thermal descriptors: LDP-LBP and LBP-LBP descriptors. The fusion method using the LDP3 operator for visible spectrum and LBP descriptor in the thermal is named FD-LDP-LBP, and FD-LBP-LBP is the fusion method using the LBP descriptor for both spectra. 

**Figure 5:**
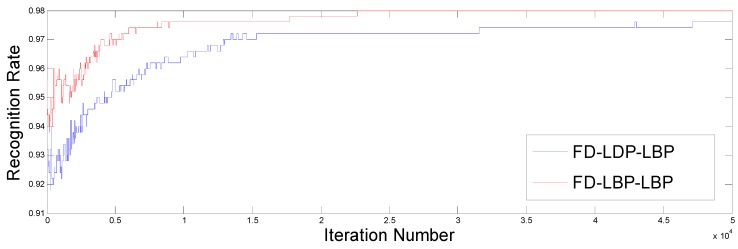
Fitness value evolution for the genetic algorithm. FD, fusion descriptor; LDP, local derivative pattern; LBP, local binary pattern.

To achieve the best genetic codes, only two subsets (the most representative) of the Equinox database are used to obtain the optimal weights. In the experiment, the GA is trained using the EA set as the gallery set and the RR set as the test set, since this set is random and represents more variability in the images (lighting conditions, expression, artefacts). The GA was applied to the gallery and test sets, finding the best possible population using the proposed fusion methodology following the steps shown in Section 3. The evolution of the genetic algorithm (fitness value) is shown in Figure 5 for both FD-LDP-LBP and FD-LBP-LBP. The optimal final population represented by 100 different individuals was found, achieving the same recognition rate for the gallery and test sets, reaching almost 98% for both fusion descriptor methods. Note that FD-LBP-LBP showed a faster convergence than FD-LDP-LBP to achieve the optimum. 

Once the GA process had been performed, the optimal population was tested using the entire Equinox database. In addition, the face recognition rate obtained using the fusion method was compared with the results obtained using the face recognition methods shown in Section 3 and the fusion results obtained from KPCA (kernel PCA) and KFLD (kernel Fisher linear discriminant) [19], wavelets and PCA [18] and discrete wavelet transform (DWT) [20]. The recognition experiments were performed using the face recognition methods applied to the gallery and test sets for the Equinox database. For each gallery and test set, the top one recognition was computed. This consists of obtaining the most similar image as a correct rate from the gallery/test sets. In addition, the average recognition rate of each result from gallery/test pairs of the entire Equinox database was used as the evaluation criteria. This is calculated as the mean of the recognition rate for each set. The results of the fusion approaches obtained from the literature [18,19,20] do not include the same gallery/test sets used in this work, because those studies often removed the case of the subject with glasses. In order to obtain a fair evaluation, the best results reported in each were considered to obtain a comparison criterion with the proposed fusion method. 

The results obtained are shown in Table 2 after applying 100 genetic codes obtained from the GA process for both fusion methods. All 100 genetic codes obtained better recognition rate results in comparison with current face recognition methods, even the worst code obtained. Table 2 shows the results from the face recognition method and only two results of the fusion descriptors, *i.e.*, the best variants for FD-LDP-LBP and FD-LBP-LBP, which were chosen to show the effectiveness of the proposed fusion method. In addition, comparing all of the results of the face recognition methods, our proposed methods increase recognition performance to 96.99%. Thus, it is possible to obtain a unique genetic code that ensures the best performance for the face recognition system with the Equinox database using FD-LDP-LBP. In addition, the FD-LBP-LBP also increases performance, achieving 95.34% for recognition and surpassing the classic visible and thermal methods. The fusion methods from the literature obtained high performance rates, achieving ~96% recognition rates; however, these fusion results are less than the results obtained from the proposed fusion method. Previous fusion techniques do not consider difficult cases, such as glasses, which further enhances our results. We believe that the high recognition rates achieved by our fusion methodology are due to the local descriptors used for the face recognition, which are robust to occlusion. Furthermore, the descriptors are not affected by lighting conditions, and the genetic algorithm efficiently combines features for the face description. For these reasons, the next experiments will not consider the evaluation of the classic fusion methods. Note that without using the proposed fusion method, the best variant for thermal face recognition is LBP, obtaining 95.32%, but in the visible case, the best method is LDP3 with only an 88.20% recognition rate. 

**Table 2:** Average recognition rate for the proposed fusion method against all face recognition methods for the Equinox database. Results in bold show the best variation. HI, histogram intersection; WLD, Weber law descriptor; GJD, Gabor jet descriptor; KPCA, kernel PCA; KFLD, kernel Fisher linear discriminant.

Method	Visible (%)	Thermal (%)
LBP_HI_80	81.60	**93.89**
LBP_HI_32	80.11	**95.32**
LDP2_X2_32	83.12	**88.98**
LDP3_HI_256	**88.20**	61.10
LDP3_X2_32	**85.22**	73.30
HOG_X2_256	73.59	**94.03**
WLD_HI_80	78.89	**84.86**
WLD_X2_80	38.05	**85.19**
GJD	**85.35**	70.07
FD-LDP-LBP	**96.99**	
FD-LBP-LBP	95.64	
Wavelets [18]	93.50	
PCA [18]	92.90	
KPCA [19]	82.70	
KFLD [19]	96.30	
Wavelets [20]	96.10	

**Figure 6:**
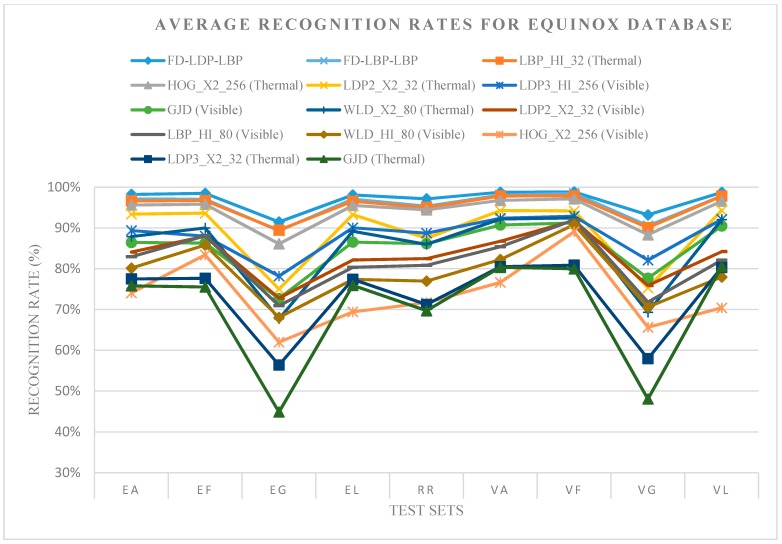
Average recognition rates for the Equinox database. Different test sets against the entire gallery sets.

Figure 6 presents an analysis of the behavior of the best variants of face recognition methods and the proposed fusion method for all Equinox galleries. The robustness of each method is evaluated by calculating the recognition rate using different conditions, such as expressions, gestures, lighting and glasses, by using different test sets from the Equinox database. Thus, for both spectra, it can be seen that FD-LDP-LBP and FD-LBP-LBP are more robust than other methods to changes in expression and the use of glasses, since they acquire visible and thermal characteristics given by the optimal weights. Additionally, considering the visible spectrum results, most of the visible methods are affected by glasses and illumination, whereas the thermal methods are more robust to expression and illumination conditions, but are affected by glasses, with LDP3 and GJD being the worst methods in this spectrum.

**Figure 7:**
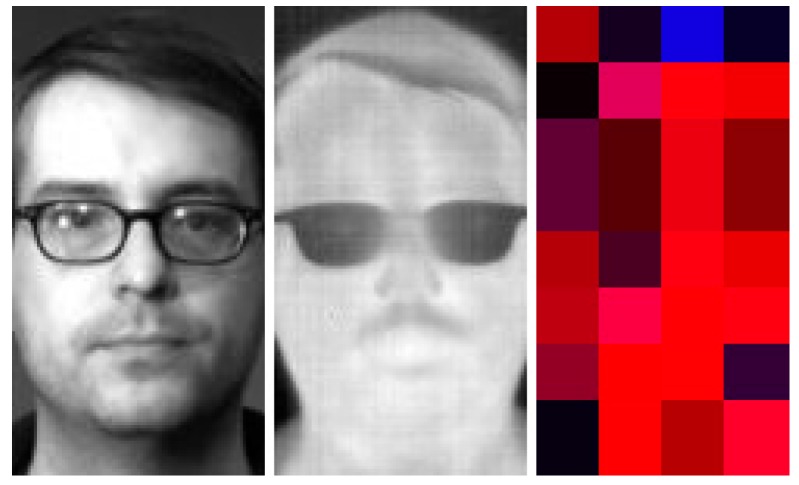
Optimal genetic code obtained for the GA.

To finish our experiment and as a means of representation, the best genetic codes obtained from FD-LDP-LBP were selected, showing that parts of the visible and thermal image of a human face are useful for face recognition process. For example, given its weight values, the best genetic code found shows that there are areas for face recognition that are more significant in the visible spectrum, and others are more important in the thermal spectrum. Figure 7 shows a face divided into 32 regions, where each region is representing by a color, blue or red, representing the information of the optimal population found with the GA. In addition, in Figure 7, it can be seen that the proposed method chose to give more relevance to the thermal regions (red parts: noose, lips and chin), because these are more invariant to changes in lighting and pose, but in the areas around the forehead and hair, it gives more relevance to visible areas (blue parts), as the thermal imaging has no information. In the next section, the two fusion descriptions, FD-LDP-LBP and FD-LBP-LBP, and the best population are used to analyze another database to validate the performance of our fusion methods.

### 5.2. Experiment 2: Fusion Method Validation Using the PUCV-VTF Database

The second experiment consist of evaluating the PUCV-VTF database with the fusion methodology *versus* the visible and thermal face recognition methods to compare and validate the proposed fusion descriptor. Firstly, the GA was not re-implemented to obtain the best population, since the best population obtained with the Equinox database was used. Secondly, the experiments were conducted using the top one criterion to obtain the recognition rate. Similar to the Equinox database, the different test sets (frown, glasses, smile and vowels) were compared with the gallery (normal) using all of the methods from Section 2 and the proposed fusion method, selecting the best results, which are shown in Table 3. From Table 3, it can be seen that the results are consistent with the Equinox database, where the fusion methodologies achieve the best results and very high recognition rates (99.01%) for FD-LBP-LBP. In addition, one fusion method, FD-LDP-LBP, and the thermal LBP variant, LBP_HI_80, obtain similar results: 98.68%. Note that thermal methods attain high performance in recognition rates with local descriptors, such as LBP, HOG and WLD. 

To show the performance of each test set *versus* the gallery set, Table 4 was created. It shows the average recognition rate of all test sets (frown, glasses, smile and vowels) *versus* the gallery set (normal). We can see that for the frown set, the two proposed fusion descriptors obtain the best performance of 98.68%, where, for example, the GJD and LDP variants in the visible and thermal perform very poorly. For the glasses set, most face recognition methods decrease their recognition rates, but the FD-LBP-LBP and the thermal LBP_HI_80 obtain the best results, remaining very robust with the use of this artefact. With the smile set, all methods are very robust to this pose variation in the thermal spectrum, where LBP, WLD, GJD and the proposed fusion methods achieve 100% recognition rates. For the vowel set, something different occurs, because here, the thermal LBP obtains 100% performance and surpasses the proposed fusion descriptor. We believe the reason is that the visible features do not give more information for the proposed fusion descriptor with the vowel set. Finally, the average results of the entire gallery/test sets are compared to identify the best performance, which is seen for the fusion descriptor methodology (FD-LBP-LBP). The result of a 99.01% recognition rate validates the proposed fusion methodology, showing high results for both the Equinox and PUCV-VTF databases. 

For both the Equinox and PUCV-VTF databases, the fusion methodology obtains high face recognition performance, achieving the best recognition rates. These results are very encouraging, since, through the use of both spectra, it is possible to combine better features than in the visible and thermal spectra separately. Thus, it can be concluded that the proposed fusion descriptor using a genetic algorithm increases the recognition rates in a visible/thermal face recognition system.

**Table 3:** Average recognition rate for the proposed fusion method against all face recognition methods for the PUCV-VTF database. Results in bold show the best variation.

Variant	Visible (%)	Thermal (%)
LBP_HI_80	91.45	**98.68**
LBP_X2_32	96.71	**98.03**
LDP2_HI_32	**94.08**	91.78
LDP2_HI_256	91.12	**93.09**
LDP3_X2_32	78.95	**83.22**
LDP3_EU_256	74.01	**86.51**
HOG_EU_256	93.09	**97.04**
HOG_X2_256	92.76	**97.70**
WLD_HI_80	90.46	**97.70**
GJD	80.26	**92.76**
FD-LDP-LBP	98.68	
FD-LBP-LBP	**99.01**	

**Table 4:** Average recognition rate for the PUCV-VTF database. Different test sets against the entire gallery sets. In bold are the best results per test set.

Variant	Average Recognition Rate-Test Sets				Average
	Frown	Glasses	Smile	Vowels	
LBP_X2_32 (Visible)	96.05	96.05	98.68	96.05	96.71
LBP_HI_80 (Thermal)	96.05	**98.68**	**100.00**	**100.00**	98.68
LDP2_HI_32 (Visible)	94.74	93.42	94.74	93.42	94.08
LDP2_HI_256 (Thermal)	89.47	92.11	97.37	93.42	93.09
LDP3_X2_32 (Visible)	65.79	77.63	86.84	85.53	78.95
LDP3_EU_256 (Thermal)	82.89	78.95	98.68	85.53	86.51
HOG_EU_256 (Visible)	88.16	92.11	96.05	96.05	93.09
HOG_X2_256 (Thermal)	96.05	97.37	98.68	98.68	93.09
WLD_HI_80 (Visible)	85.53	85.53	97.37	93.42	90.46
WLD_HI_80 (Thermal)	96.05	97.37	**100.00**	97.37	97.70
GJD (Visible)	60.53	69.74	97.37	93.42	80.26
GJD (Thermal)	92.11	82.89	**100.00**	96.05	92.76
FD-LDP-LBP	**98.68**	97.37	**100.00**	98.68	98.68
FD-LBP-LBP	**98.68**	**98.68**	**100.00**	98.68	**99.01**

## 6. Conclusions

In this study, a new fusion methodology to combine visible and thermal descriptors for face recognition systems was presented. The proposed fusion methodology was evaluated using the standard visible and thermal database, Equinox, and a new database, entitled PUCV-VisibleThermal-Face, created to validate the proposed fusion method and to be used in further research on visible and thermal face recognition. In addition, this study compared the recognition rates of current face recognition methods, such as local derivative patterns, local binary patterns, Weber local descriptors, histograms of oriented gradients and Gabor jet descriptors, and classic fusion techniques used commonly in the literature; against the proposed methodology of fusion descriptors using a genetic algorithm to find the best population and, thus, to achieve an optimal face recognition rate. 

To achieve the optimal population for the fusion method, the methodology was tested using a part of the Equinox database, achieving a fitness convergence of 98% recognition rate. Thus, with the optimal population, the fusion descriptor was evaluated using the entire Equinox database and the associated methodology [7,12]. The results show that the two fusion descriptors used (FD-LDP-LBP and FD-LBP-LBP) obtain high performances, surpassing the visible and thermal results of the current face recognition methods and other classic fusion approaches used in the literature. In addition, observing the results obtained by the fusion descriptor, the methodology is very robust to changes in illumination and expression, because it combines thermal and visible information, allowing it to choose optimal face areas where one spectrum is more representative than the other. However, when the individual uses an artefact, such as glasses, and although the fusion descriptor results are superior to the visible/thermal face recognition methods, the performance decreases slightly, since the optimal weights are statically obtained with images that randomly contain glasses and no glasses, as reported in [18,20]. We believe that this can be solved by increasing the variability of the images used to obtain the optimal population (more examples to increase the fitness values/recognition rates).

The fusion methodology was validated using the average recognition rate obtained from the evaluation of current visible/thermal face recognition rates and the PUCV-VTF database. The results show the high performance obtained by the fusion descriptor (FD-LBP-LBP), achieving a recognition rate of 99.01%. This result validates the fusion method, as it shows that the fusion descriptor operates with high recognition rates and is robust to images with different variabilities, such as expressions (frown, smile), gestures and artefacts, such as glasses. 

The final conclusion on the fusion descriptors is that combining visible and thermal descriptors increases the performance of the face recognition system. The analysis of the fusion descriptor and the evaluation with the two databases shows that using a genetic algorithm to optimize the recognition rates gives an optimal population and leads to the best performance, due to the selection of the optimal weights and, thus, obtaining the best possible combination of visible and thermal features. We conclude that the proposed fusion method is very robust to variations and artefacts and obtains high recognition rates. However, as a future study, these results could be increased with new techniques based on evolutionary algorithms [29], or with the combination of new face recognition descriptors [30,31], or new texture classification methods published in recent literature. 
